# Collaborative Neural Network Algorithm for Event-Driven Deployment in Wireless Sensor and Robot Networks

**DOI:** 10.3390/s20102779

**Published:** 2020-05-13

**Authors:** Yaoming Zhuang, Chengdong Wu, Hao Wu, Zuyuan Zhang, Yuan Gao, Li Li

**Affiliations:** 1 Faculty of Robot Science and Engineering, Northeastern University, Shenyang 110819, China; wuchengdong@ise.neu.edu.cn; 2 Engineering Faculty, University of Sydney, Sydney, NSW 2006, Australia; hawu1598@uni.sydney.edu.au; 3 School of Compute Science, University of Oklahoma at Norman, Norman, OK 73070, USA; Zuyuan.Zhang-1@ou.edu; 4 College of Information Science and Engineering, Northeastern University, Shenyang 110819, China; gaoyuan@stumail.neu.edu.cn; 5 JangHo School of Architecture, Northeastern University, Shenyang 110819, China; lili1118@mail.neu.edu.cn

**Keywords:** event-driven deployment, collaborative neural network, maximum entropy function, multiple constraints, wireless sensor and robot networks

## Abstract

Wireless sensor and robot networks (WSRNs) often work in complex and dangerous environments that are subject to many constraints. For obtaining a better monitoring performance, it is necessary to deploy different types of sensors for various complex environments and constraints. The traditional event-driven deployment algorithm is only applicable to a single type of monitoring scenario, so cannot effectively adapt to different types of monitoring scenarios at the same time. In this paper, a multi-constrained event-driven deployment model is proposed based on the maximum entropy function, which transforms the complex event-driven deployment problem into two continuously differentiable single-objective sub-problems. Then, a collaborative neural network (CONN) event-driven deployment algorithm is proposed based on neural network methods. The CONN event-driven deployment algorithm effectively solves the problem that it is difficult to obtain a large amount of sensor data and environmental information in a complex and dangerous monitoring environment. Unlike traditional deployment methods, the CONN algorithm can adaptively provide an optimal deployment solution for a variety of complex monitoring environments. This greatly reduces the time and cost involved in adapting to different monitoring environments. Finally, a large number of experiments verify the performance of the CONN algorithm, which can be adapted to a variety of complex application scenarios.

## 1. Introduction

Event-driven deployment is a new research area in wireless sensor and robot networks (WSRNs) [1,2,3,4]. Traditional sensor and robot network deployment research is mostly focused on the deployment of a single type of sensor in a single scenario [3]. In practical applications, many different types of sensors need to be deployed according to various monitoring scenarios, in order to meet the environmental monitoring requirements [5]. For example, in the indoor monitoring of large buildings, it has been observed that the interior environment of these buildings is very complex. There are many parameters that need to be monitored. The temperature and smoke density need to be tracked in the fire monitoring module, the vibration and infrared radiation need to be measured in the security module, and the identity of the person entering a sensitive area needs to be determined in the privacy monitoring module. Only deploying a single type of sensor cannot meet the monitoring needs of the networks [6]. At the same time, how to properly deploy various types of sensors in large and complex environments is an urgent problem that needs to be solved.

Koutsougeras first proposed event-driven sensor deployment [7], taking into account a realistic consideration of the probability density for events to be sensed, termed event-driven coverage. Event-driven deployment is a novel deployment problem which is different from traditional deployment, where the deployment costs of sensors, total budget, and compound events are not considered. The purpose of the event-driven deployment application is to reasonably apply a variety of different types of sensor resources. Event-driven deployment is the basis of event coverage. The quality of event-driven deployment determines the monitoring effect of event coverage. There have been a series of studies on the event coverage problem. Cardei defined single (or atomic) events or compound events for event detection in wireless sensor networks [8]. Furthermore, Gao proposed the event detection problem in heterogeneous sensor networks [9]. On the basis of event detection, Gao considered the problem of event coverage under a single cost constraint [6]. Additionally, Zhou applied event coverage detection and event source determination in underwater wireless sensor networks [10].

In practical applications, wireless sensor and robot networks often work in complex and extreme environments [11,12,13]. When sensors are deployed in a complex environment, they will be subject to various constraints, such as distance, time, cost, and confidence constraints [14,15,16]. For example, in border monitoring, the deployment of sensors is prohibited across national boundaries. The deployment of sensors will be subject to distance constraints. In battlefield monitoring, the deployment of sensors to sensitive areas must be completed within the specified time; otherwise, it may impact the chance of winning a battle. The deployment of sensors will also be subject to time constraints. Moreover, in the monitoring of large and complex buildings, a large number of sensors need to be deployed. However, the total project budget is likely limited. Therefore, the deployment of sensors will be subject to cost constraints; for intrusion detection, the accuracy of the target intrusion needs to be judged to be over 90%. The deployment of sensors will be constrained by the confidence.

The traditional wireless sensor and robot network deployment algorithm is only suitable for a certain kind of specific application scenario. When this scenario is changed or applied to other environments, the monitoring performance will be greatly compromised. For example, in community security monitoring, the scale of the networks is small. The requirements for time, distance, and cost constraints are low. However, in forest fire monitoring, the scale of the networks is large, so the requirements for time constraints are lower, but the requirements for distance and cost constraints are higher. For battlefield frontiers and volcanic monitoring, the networks are complex and large-scale, so the requirements for time and distance constraints are high, while the requirements for cost constraints are low. Therefore, different scenarios have different constraints and network sizes. In the face of increasing application scenarios and various constraints, traditional wireless sensor and robot network deployment methods cannot meet the requirements of various application scenarios. Event-driven deployment is based on different monitoring events and network constraints and uses different types of sensors to provide the optimal deployment scheme in complex and dangerous environments. First, the traditional deployment problem only focuses on the quality of network deployment, and does not consider the quality of event monitoring, such as confidence. Secondly, the traditional deployment problem does not consider the optimal allocation scheme of sensor resources based on different monitoring events. Finally, the traditional deployment problem does not consider multiple constraints in the complex wireless sensor and robot network environment.

The traditional event-driven deployment algorithm is only applicable to a single type of monitoring scenario, so cannot effectively adapt to different types of monitoring scenarios at the same time. However, wireless sensor and robot networks often work in a variety of complex and dangerous environments. As a result, it is difficult to obtain a large amount of sensor data and environmental information in practical applications.

Based on the above analysis, it is necessary to propose a network deployment method that can adaptively provide an optimal deployment solution for a variety of complex scenarios with multiple constraints. Our contributions can be summarized as follows:

- It is the first time that complex event-driven deployment problems are solved by neural network methods. By using collaborative neural networks, the event-driven deployment algorithm can be adapted to different application scenarios to meet a variety of monitoring requirements;
- For the first time, a multi-constrained event-driven deployment model based on the maximum entropy function is proposed. This model transforms the complex event-driven deployment problem into two single-objective sub-problems. Both of the sub-problems are continuously differentiable. This model overcomes the limitation that the objective function of the traditional min–max method is non-differential;
- It is the first time that the difficulty of obtaining a large amount of sensor data and environmental information in a complex and dangerous monitoring environment is effectively overcome. Unlike traditional deployment methods, the collaborative neural network (CONN) algorithm can adaptively provide an optimal deployment solution for a variety of complex monitoring environments. This greatly reduces the time and cost involved in adapting to different monitoring environments.

The rest of this paper is arranged as follows: related works are described in Section 2; the compound event-driven deployment model is proposed in Section 3; the compound event-driven deployment algorithm based on the collaborative neural network strategy is proposed in Section 4 and evaluated in Section 5; and Section 6 concludes this paper.

## 2. Related Works

In research on deployment issues in wireless sensor and robot networks, the primary goal is for event-driven deployment to provide effective deployment schemes according to the monitoring environment [17,18]. Janakiram first proposed the concept of event detection [19]. Based on a proposed event detection model, in [20,21], the issue of energy efficiency for event monitoring is highlighted. In [9], the authors identify the problem of the optimal data transmission mechanism in event monitoring. In [6], the authors take into account the deployment costs involved in event monitoring. Moreover, in the practical application of event monitoring, event-driven deployment will not only be subject to a single energy or cost constraint, but will also be affected by the time, distance, cost, confidence, and other complex constraints. In [10], the authors apply the routing tree and weighted graph method to detect and determine events in underwater sensor networks. It requires a lot of manpower and many material resources to obtain a large amount of sensing data in underwater sensor networks.

The deployment methods can be divided into deterministic deployment and random deployment. Deterministic deployment is often used when sensors are expensive or the location of the nodes will have a significant impact on the network. For example, the deployment methods of distance measuring sensors, underwater ultrasonic sensors, and camera sensors generally involve deterministic deployment. In [22], the authors propose a k-equivalent radius enhanced virtual force algorithm for achieving an uneven regional coverage optimization for different requirements. In [23], the authors present an optimized strategy in which sensor deployment begins from the center of the target region by adding an external central force to avoid covering holes or unusual structures. Random deployment is applied in harsh environments, such as battlefields or disaster-prone areas, where people or mechanical equipment cannot enter and can only be deployed by means of aircraft spreading. In [24], the authors propose centralized and distributed deployment algorithms to identify the optimal sensor deployment to maximize the sensing coverage with specific energy constraints in wireless sensor and robot networks. Currently, the deployment methods used in wireless sensor and robot networks are not suitable for a variety of monitoring requirements in different application scenarios.

Event-driven deployment will be subject to various constraints in a complex environment [25,26]. In [27], the authors propose a new min-max problem in networks with a maximization framework to deal with multiple constraints in wireless sensor networks. In [28], the authors propose a mathematical model for a novel quality-of-service (QoS) routing determination method to optimize a multi-constrained quality-of-service multipath routing approach for multimedia sensor networks. In [29], a distributed anycast algorithm, ARMCA, inspired by the swarm intelligence of ants, is proposed to solve energy and resource constraints in the deployment of wireless sensor networks. In summary, the above methods employed to solve multi-constraint problems are very complex and often specific to a local optimal solution.

With the maturity of neural network methods, more and more research on neural network methods is being applied to wireless sensor networks [30,31,32]. In [33], the authors propose the concept of collaborative neural networks. The collaborative neural network is an artificial neural network consisting of two or more multiple neural networks. Each neural network influences each other in the operation to obtain the solution of the original problem. In [34], the authors propose collaborative recurrent modular neural networks for a constrained optimization. In [35], the authors prove that neural networks have the characteristics of global convergence. In [36], the authors propose a collaborative recurrent neural network for dynamic recommender systems. The paper [37] presents private collaborative neural network learning by combining differential privacy and secure multi-party computation with machine learning. In order to achieve better monitoring results, in [38], the authors propose a secure sensor network localization method based on neural networks. While implementing the positioning function, in [32], the authors use the recurrent neural network and Kalman filtering method to achieve the real-time tracking of targets in wireless sensor networks. The paper [31] proposes to take advantage of wireless sensor networks and neural networks to effectively monitor falls among the elderly and locate the position of the fall. The paper [39] proposes that a wireless sensor network-based multi-sensor system and artificial neural network can be used to monitor the occurrence and scale of forest fires in real time. In [40], the authors apply ZigBee-based mobile wireless sensor networks and an artificial neural network to achieve the effective monitoring and classification of animal behavior.

The above methods often require a large amount of sensor data and environmental information in order to achieve a better monitoring performance. In practical applications, wireless sensor and robot networks are usually deployed in complex and dangerous environments, where it is difficult to obtain a large amount of sensor data and environmental information. Additionally, the above methods do not take into account the various constraints that wireless sensor and robot networks are exposed to in practical applications.

## 3. Compound Event-Driven Deployment Model Based on the Maximum Entropy Function

### 3.1. Main Idea

In this section, a compound event-driven deployment model based on the maximum entropy function is proposed to reasonably deploy diverse types of sensors, in order to obtain a better monitoring performance in multi-constrained wireless sensor and robot networks.

The multi-constrained event-driven deployment model based on the maximum entropy function transforms the complex event-driven deployment problem into two continuously differentiable single-objective sub-problems, which effectively solves the problem of the objective function of the min–max method being non-differential.

### 3.2. Problem Formulation

In order to concisely formulate problems, preliminaries are defined. A variety of complex conditions in complex and dangerous environments are defined as inequality constraint functions. Without a loss of generality, the compound event-driven deployment problem with multiple constraints is defined as follows:

(1) $$\begin{array}{ll} \max F(\alpha )=c_{i}f_{i}(\alpha_{1},\alpha_{2},\ldots ,\alpha_{n}) & (i=1,2,\ldots ,p),\\ s.t.\;f_{j}(\alpha )=g_{j}(\alpha_{1},\alpha_{2},\ldots ,\alpha_{n})\leq 0 & \left(j=1,2,\ldots ,q\right) \end{array}$$

where F(α) is the event-driven deployment function; *i* is the number of objective functions; *j* is the number of constraint functions; $c_{i}$ is the confidence coefficient, where $c_{i}\geq 0$, and reflects the monitoring accuracy of different types of sensors; $\alpha =(\alpha_{1},\alpha_{2},\ldots ,\alpha_{n})$ represents the different types of sensors, where $\alpha =(\alpha_{1},\alpha_{2},\ldots ,\alpha_{n})\in T\subset R^{n}$ is an *n*-dimensional decision vector; $T=\{\alpha \in R^{n}|d_{i}\leq \alpha \leq u_{i},i=1,2,\ldots ,p\}$ is the target space, where $d_{i}\in R$ and $u_{i}\in R$ denote the lower bound and upper bound of $\alpha_{i}$, respectively; $f_{j}(\alpha )$ represents inequality constraints; and $g_{j}(\alpha )$ denotes the inequality constraint function. The *g*(*α*) functions are all positive values that represent different constraints in the practical application, such as distance, time, and cost constraints. $\Omega =\{\alpha |\alpha \in T,g_{i}(\alpha )\leq 0,i=1,\ldots ,p\}$ is the feasible domain. The points in the feasible domain are feasible solutions.

The norm of Formula (1) is

(2) $${\left\|NF\right\|}_{\infty }=\underset{1\leq i\leq p}{\max}\left\{c_{i}f_{i}(\alpha )\right\}\;\;\;(i=1,2,\ldots ,p),$$

where $N=diag(c_{1},c_{2},\ldots ,c_{p})$.

In order to improve the accuracy of the calculation, the compound event-driven deployment model introduces reference points $f_{i}{}^{*}$. The reference points introduced are vectors corresponding to the number of objects. That is, each component corresponds to the optimal value of each object [41]. The compound event-driven deployment model that introduces reference points can be expressed as

(3) $$\begin{array}{ll} \max\{c_{i}f_{i}(\alpha )-f_{i}{}^{*}\} & (i=1,2,\ldots ,p).\\ s.t.\;\;f_{j}(\alpha )=g_{j}(\alpha_{1},\alpha_{2},\ldots ,\alpha_{n})\leq 0 & \left(j=1,2,\ldots ,q\right) \end{array}$$

The maximum entropy function of Formula (1) is as follows [42,43]:

(4) $$F_{e}(\alpha )=\frac{1}{e}\ln\sum_{i=1}^{p}\exp(ef_{i}(\alpha )),e>0.$$

Formula (4) plays a central role in the present development and is referred to as the aggregate function. It can be shown that $F_{e}(\alpha )$ will approach $\underset{i}{\max}\{f_{i}(\alpha )\}$ as the parameter *e* tends to infinity. In fact, $F_{e}(\alpha )$ denotes a uniform approximation of the problem functions in the limit of *e* approaching infinity.

The properties of the maximum entropy function are as follows [42,43].

Property 1: When e→∞, for any $\alpha \in R^{n}$, $F_{e}(\alpha )$ converges to η(α), where $\eta (\alpha )=\underset{1\leq i\leq p}{\max}\left\{f_{i}(\alpha )\;\right\}\;\;\;(i=1,2,\ldots ,p)$.

Before describing the algorithm, we examine the extent to which $F_{e}(\alpha )$ approximates η(α). To this end, we can write $F_{e}(\alpha )$ in terms of η(α) as

(5) $$F_{e}(\alpha )=\eta (\alpha )+\frac{1}{e}\ln\sum_{i=1}^{p}\exp\{e[f_{i}(\alpha )-\eta (\alpha )]\}.$$

From the definition of η(α), it immediately follows that

(6) $$f_{i}(\alpha )-\eta (\alpha )\leq 0\;i=1,2,\ldots ,p,\;$$

in which the equality must hold for at least some *i* so that the second term in Formula (5) lies between 0 and $\frac{1}{e}\ln(p)$.

Property 2: For any e>0, $\exists \delta (\alpha )=F_{e}(\alpha )-\eta (\alpha )$, $0\leq \delta (\alpha )\leq \frac{1}{e}\ln(p)$, where *p* is the number of problem functions. From Property 2, it can be seen that $F_{e}(\alpha )$ is always larger than η(α) and differs from η(α) by a value of less than $\frac{1}{e}\ln(p)$, which is negligible when *e* is sufficiently large.

Property 3: $F_{e}(\alpha )$ is a differentiable convex function.

According to Property 1, the multi-constrained compound event-driven deployment problem in Formula (1) can be solved by the following multi-constrained single-objective event-driven deployment sub-problem:

(7) $$\begin{array}{ll} \max F_{e}(\alpha ,c)=\frac{1}{e}\ln\sum_{i=1}^{p}\exp(ec_{i}(f_{i}(\alpha )-f_{i}{}^{*})) & (i=1,2,\ldots ,p).\\ s.t.\;f_{j}(\alpha )=g_{j}(\alpha_{1},\alpha_{2},\ldots ,\alpha_{n})\leq 0 & \left(j=1,2,\ldots ,q\right) \end{array}$$

Formula (7) is a function maximization problem relating to the independent variables α and c. This method transforms the complex compound event-driven deployment problem into a continuously differentiable single-objective sub-problem which does not introduce new variables and does not increase the number of constraints. Therefore, this method has a higher operating efficiency than the traditional event-driven deployment method [6].

According to the single-objective sub-problem above, suppose $c^{*}=[c_{1},c_{2},\ldots ,c_{n}]$ is the optimal confidence coefficient of Formula (7). Therefore, the Lagrange equation of Formula (7) is as follows [44]:

(8) $$L(\alpha ,\gamma ,c^{*})=F_{e}(\alpha ,c^{*})+\gamma^{T}g(\alpha ).$$

In Formula (8), $\gamma \in R^{n}$ is a vector of the Lagrange multiplier and $F_{e}(\alpha ,c^{*})$ is a convex programming problem. According to the Karush–Kuhn–Tucker condition, $\alpha^{*}$ is the optimal solution of Formula (7) if, and only if, there is a Lagrange multiplier vector $\gamma^{*}$, which makes $\left(\alpha^{*},\gamma^{*}\right)$ satisfy

(9) $$\left\{ \begin{array}{l} \frac{\partial L}{\partial \alpha }=0\\ g(\alpha )\leq 0,\gamma \geq 0,\gamma^{T}g(\alpha )=0 \end{array}\right..$$

In Formula (9),

(10) $$\frac{\partial L}{\partial \alpha }=\frac{c_{i}}{\sum_{i=1}^{p}\exp(ec_{i}(f_{i}(\alpha )-f_{i}^{\prime \prime }))}\sum_{i=1}^{p}\exp(ec_{i}(f_{i}(\alpha )-f_{i}^{\prime \prime }))\frac{\partial f_{i}(\alpha )}{\partial \alpha }+\gamma_{j}\sum_{j=1}^{q}\frac{\partial g_{j}(\alpha )}{\partial \alpha },$$

where

(11) $$\frac{\partial L}{\partial \alpha }=\gamma_{j}\sum_{j=1}^{q}\frac{\partial g_{j}(\alpha )}{\partial \alpha }+\frac{c_{i}\sum_{i=1}^{p}\exp(ec_{i}(f_{i}(\alpha )-f_{i}^{*}))\frac{\partial f_{i}(\alpha )}{\partial \alpha }}{\sum_{i=1}^{p}\exp(ec_{i}(f_{i}(\alpha )-f_{i}^{*}))}.$$

According to Property 2, for any c(c>0), when $\alpha \in R^{n}$,

(12) $$\underset{1\leq i\leq p}{\min}\{c_{i}(f_{i}(\alpha )-f_{i}^{*})\}\geq F_{e}(\alpha ,c).$$

The optimal confidence coefficient can be calculated by the following single-objective event-driven deployment method:

(13) $$\begin{array}{l} \max F_{e}(\alpha ,c)=\frac{1}{e}\ln\sum_{i=1}^{p}\exp(ec_{i}(f_{i}(\alpha )-f_{i}^{*}))\\ s.t.\;\;c\geq 0 \end{array}$$

Therefore, the multi-constrained compound event-driven deployment problem is deconstructed into two sub-problems of Formulas (7) and (13). Both of the sub-problems are continuously twice differentiable and can be solved by neural networks. In the next section, a collaborative neural network strategy is used to iteratively solve the two sub-problems. Finally, the Pareto optimal solution for the multi-constrained compound event-driven deployment problem is obtained.

## 4. Compound Event-Driven Deployment Algorithm Based on the Collaborative Neural Network Strategy

### 4.1. Main Idea

The collaborative neural network is an artificial neural network consisting of two or more multiple neural networks. Compared to a single neural network or an integrated neural network, the advantages of a collaborative neural network are as follows: (1) It is suitable for large-scale and complex application scenarios; (2) It adaptively allocates each neural network module to assess sub-problems; and (3) It permits global access to the optimal solution of the original problem [33,34]. Therefore, the collaborative neural network can be applied to solve a large-scale and complex multi-constraint compound event-driven deployment problem.

### 4.2. The Neural Network Strategy for a Sub-Problem

Based on the maximum entropy function, the multi-constrained compound event-driven deployment problem is deconstructed into two sub-problems of Formulas (7) and (13). These two sub-problems are continuously twice differentiable. Next, two neural networks are applied to solve these two sub-problems. These two neural networks interact to efficiently solve the multi-constrained compound event-driven deployment problem, forming a collaborative neural network.

The main feature of the collaborative neural network is that there is a continuous path which causes the optimization process to start from the starting point and eventually converge to the optimal solution. The state equation of the collaborative neural network can be expressed as

(14) $$\frac{d}{ds}\left(\begin{array}{l} \alpha \\ \beta \end{array}\right)=\xi \left(\begin{array}{l} -\nabla_{\alpha }f(\alpha )-\nabla_{\alpha }(\alpha {)}^{T}\beta \\ -\beta +(\beta +g(\alpha ){)}^{+} \end{array}\right),$$

where $\nabla_{\alpha }g{\left(\alpha \right)}^{T}=(\nabla_{\alpha }g_{1}(\alpha ),\ldots ,\nabla_{\alpha }g_{q}(\alpha ){)}^{T}$, and $\nabla_{\alpha }g_{j}(\alpha )$ is the gradient of $g_{j}$ for *i* = 1, …, *q*.

In Formula (14), ξ is a positive constant, and $\alpha \in R^{n}$, $\beta \in R^{q}$, ${\left(\beta \right)}^{+}={\left[{\left(\beta_{1}\right)}^{+},\ldots ,{\left(\beta_{q}\right)}^{+}\right]}^{T}$, with ${\left(\beta_{j}\right)}^{+}=\max\{0,\alpha \}$.

Sub-problem (7) can be solved by the neural network:

(15) $$\frac{d}{ds}\left(\begin{array}{l} \alpha \\ \beta \end{array}\right)=\xi \left(\begin{array}{l} -\nabla_{\alpha }F_{e}(\alpha ,c)-\nabla_{\alpha }g(\alpha {)}^{T}\beta \\ -\beta +(\beta +g(\alpha ){)}^{+} \end{array}\right).$$

Sub-problem (13) can be solved by another neural network:

(16) $$\frac{dc(s)}{ds}=\xi (c-\nabla_{c}F_{e}(\alpha ,c))-c.\;$$

In Formula (16), ξ is a positive constant, $c\in R^{p}$.

### 4.3. Compound Event-Driven Deployment Algorithm Based on the Collaborative Neural Network Strategy

In this section, a compound event-driven deployment algorithm based on the collaborative neural network strategy is proposed. Firstly, the multi-constrained compound event-driven deployment problem is transformed into two single-objective sub-problems by the maximum entropy function. Then, assuming an initial confidence coefficient, the collaborative neural networks are applied to solve two single-objective sub-problems. If the solution for either of these sub-problems is the Pareto optimal solution to the multi-constrained compound event-driven deployment problem, then the algorithm terminates and outputs; otherwise, another neural network is used to update the confidence coefficient sub-problem. For the new confidence coefficient, the neural network is used to re-solve the single-objective convex programming sub-problem. The process is repeated until the Pareto optimal solution to the event-driven deployment problem is found. Compared with the traditional compound event-driven deployment method, the algorithm based on the collaborative neural network strategy is rapidly convergent and globally optimal.

**Figure 1:**
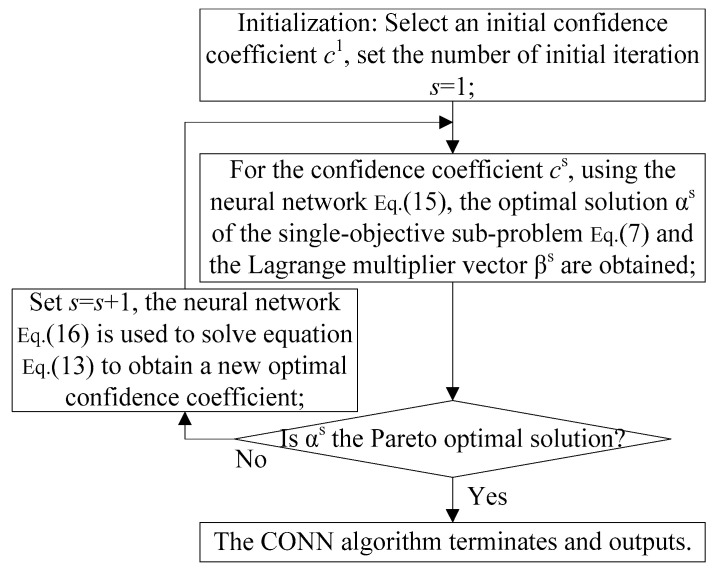
The flow diagram of the collaborative neural network (CONN) algorithm.

These two sub-problems are continuous convex single-objective problems. Therefore, two neural networks are applied to solve these two sub-problems. These two neural networks constitute a collaborative neural network. The flow diagram of the CONN algorithm is presented in Figure 1 for clarity.
**Algorithm 1** Compound Event-Driven Deployment Algorithm Based on the Collaborative Neural Network Strategy.1: Initialization: An initial confidence coefficient $c^{1}$ is selected, and the number of initial iterations s=1 is set;2: For the confidence coefficient $c^{s}$, using Neural Network (15), the optimal solution $\alpha^{s}$ of the single-objective Sub-problem (7) and the Lagrange multiplier vector $\beta^{s}$ are obtained. If $\alpha^{s}$ is the Pareto optimal solution for the multi-constrained compound event-driven deployment problem, the algorithm terminates and outputs; otherwise, the process skips to step 3;3: After obtaining $\alpha^{s}$ and $\beta^{s}$ in step 2, Neural Network (16) is used to solve Formula (13) to obtain a new optimal confidence coefficient;4: The number of iterations is set and the process skips to step 2.

### 4.4. The Complexity and Convergence Analysis of the Algorithm

Xia [35] has proved that neural networks are globally convergent. The global optimal solution of Formulas (7) and (13) can be obtained by the CONN algorithm. Therefore, the CONN algorithm is globally convergent.

The original problem of Formula (7) is the same as that of the min-max method. Therefore, the complexity of Formula (7) is the same as that of the min–max method, which is $O(n^{3})$ (*n* is the dimension of the independent variable) [45]. By introducing a new variable, the min-max method realizes the conversion of the model and ensures that the objective function is continuously differentiable. However, the min-max method also adds an independent variable β and *p* additional constraints, which increases the computational complexity of the algorithm. Compared with the min-max method used for solving complex event-driven deployment problems, the algorithm in this paper does not introduce new variables and does not increase the number of constraints. At the same time, the neural network method has obvious advantages in solving the multi-constrained convex programming problem. Therefore, the CONN algorithm is superior in terms of the time complexity.

## 5. Performance Evaluation

In the experiment, the CONN algorithm was evaluated in various deployment environments. In order to evaluate the performance of the CONN algorithm, the CONN algorithm was used to complete event-driven deployment in multi-constrained WSRNs. In the experiment, five types of sensors were used, which had a different perceived radius, deployment time, cost, and confidence values. In event-driven deployment, not only do the distance, time, cost, and minimum confidence constraints need to be met, but the quality of event-driven deployment is also required. In the performance comparison experiment, the CONN algorithm was compared with ASMP, EPDS, and OCQ-Max-fit algorithms in the same experimental environment [6,25,26]. First, in order to compare the adaptability of the CONN algorithm in different deployment areas, the CONN algorithm was compared with the other three algorithms in the area from 100 × 100 to 1000 × 1000, respectively. Next, in order to verify the performance of the CONN algorithm under different deployment budgets, the CONN algorithm was compared with the other three algorithms with the budget from 100 to 600, respectively. Finally, in order to test the adaptability of the CONN algorithm to the number of sensor types, the CONN algorithm was compared with the other three algorithms with different numbers of sensor types, respectively.

### 5.1. Environment Settings

Matlab2014a was used to perform the experiments. The experiments were operated on a desktop with an Intel(R) Core(TM) i7-6700 CPU @ 3.40 GHz, an 8-GB memory, and the 64-bit Windows 10 system. In the experiments, there were five types of sensors. The confidence coefficients of the five types of sensors were 0.35, 0.20, 0.15, 0.05, and 0.10, respectively. Each type of sensor had a different perceived radius, deployment time, deployment cost, and confidence, according to its own properties. The parameters of these sensors are presented in Table 1. Experiments were performed for four different cases, in order to assess the performance in different situations.

### 5.2. Experimental Evaluation

The experiments were performed to analyze the efficiency of the CONN algorithm in multi-constrained WSRNs. The constraints of distance, time, cost, and minimum confidence are listed in Table 2 for four different cases. The experimental results and analysis are presented in the following section.

In Table 1, the meanings of the abbreviations are as follows. R: perceived radius (R/m); T: deployment time (T/s); C: cost (C); Conf: confidence.

In Table 2, the meanings of the abbreviations are as follows. D: distance constraints; T: time constraints; C: cost constraints; MIN: minimum confidence constraints; Ratio: coverage ratio; Time: computation time.

**Figure 2:**
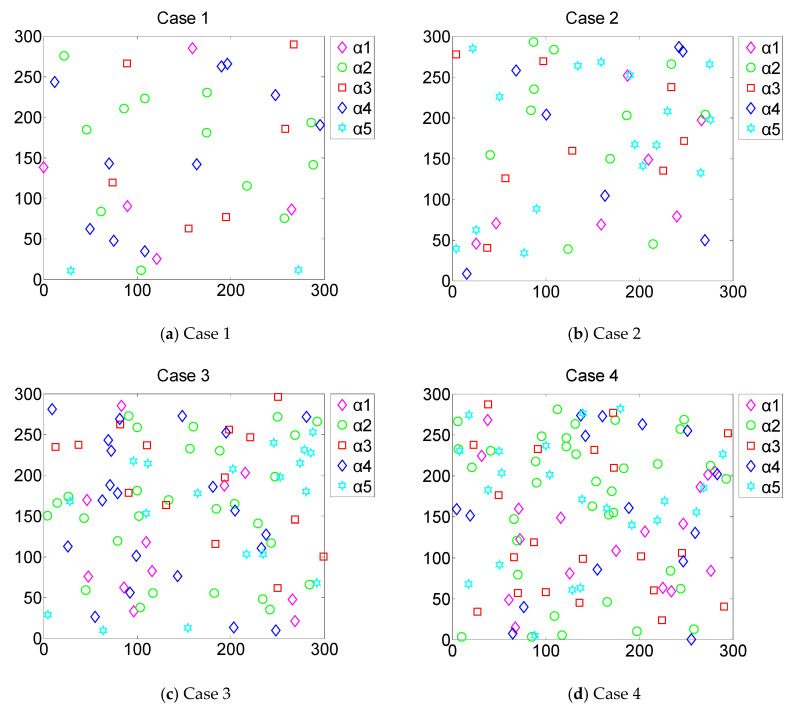
The CONN algorithm was applied for the deployment of five different types of sensors under four different network constraints in Case 1 to Case 4.

Figure 2 shows that the CONN algorithm was used for the deployment of five types of sensors under four different constraints. It can be seen that the minimum confidence constraint from case 1 to case 4 continuously rose. The monitoring requirements for the network also increased. Therefore, it is necessary to deploy additional diverse types of sensors in the networks.

At the same time, each case was also subject to different constraints of distance, time, and cost. The four cases represent four different network constraints, which are listed in Table 2. These four cases were extracted separately for a more intuitive effect. As the number of sensors in the network increased, the sensor deployment in the network gradually became uniform. The coverage holes in the network were also gradually reduced. Two neural networks in the CONN algorithm interacted and corrected each other. The event-driven deployment was continuously optimized in the network. When the number of sensors in the network was large, the CONN algorithm could still provide effective event-driven deployment to the network.

In case 1, the network is subject to strict distance constraints, time constraints, and cost constraints. The number of sensors that can be used in the network is limited. Therefore, the deployment performance of the network is unsatisfactory. The final coverage ratio is only 42.6%. In case 2, as the constraints in the network increase, the number of sensors available in the network begins to increase. The deployment performance of the network begins to improve, reaching 66.2%. In case 3, as the number of sensors available increases further, the coverage holes in the network are significantly reduced. Meanwhile, the deployment of the network tends to be uniform, reaching 89.5%. In case 4, as the number of sensors available increases, the network deployment tends to be saturated. The increase of the coverage ratio has gradually slowed down. The coverage ratio finally reaches 96.7%. Although the number of sensors in the network tends to be saturated, the CONN algorithm can continue to converge to the optimal solution under the current network conditions, which means that the network deployment tends to be reasonable. It can be seen that the CONN algorithm can reasonably deploy multiple types of sensors according to different network constraints.

**Figure 3:**
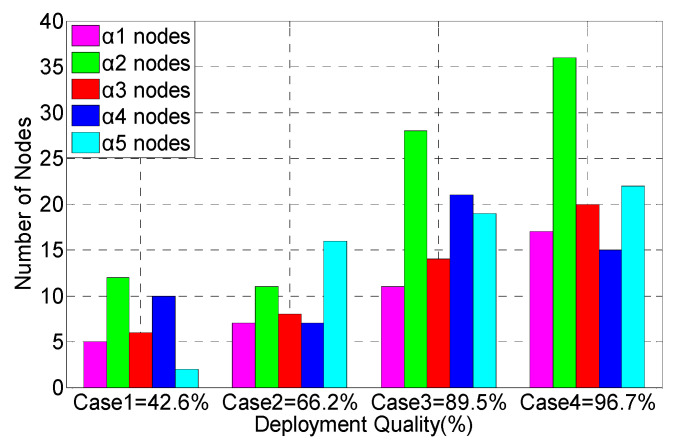
The sensor resource allocation of four cases and the event-driven deployment performance of the CONN algorithm.

Figure 3 shows the sensor resource allocation of these four cases and the event-driven deployment performance. Each of the four cases has different distance, time, cost, and confidence constraints. It can be seen that the minimum confidence requirement for case 1 to case 4 is constantly rising. The monitoring requirements of the network are also constantly improving. Case 1 and case 3 are not only subject to strict cost constraints, but also constrained by distance, time, and confidence. Therefore, lower-cost RFID (Radio Frequency Identification) sensors and Bluetooth sensors are used more frequently. It can be seen that case 2 is not only limited by strict time constraints, but also by distance, cost, and confidence constraints. Therefore, the pyroelectric sensors with a shorter deployment time are widely used. In case 4, the requirement of the confidence for the network deployment is higher. Therefore, the camera sensors are used more frequently. In fact, it is the highest camera sensor usage in the four cases. However, in case 4, the network is also subject to distance, time, and cost constraints. Although camera sensors have the highest monitoring accuracy, the deployment time is the longest. The deployment cost is also the highest. Therefore, in order to meet a variety of complex constraints, lots of RFID sensors and pyroelectric sensors are also applied in network deployment. The experimental results show that the CONN algorithm can efficiently allocate sensor resources in multiple constraint sensor and robot networks.

### 5.3. Comparison with the ASMP Algorithm in Terms of the Deployment Intensity

This section presents a comparison of the CONN and ASMP algorithm in terms of the deployment intensity [25]. The ASMP algorithm is a multiplier method based on an active-set strategy used to solve the trade-offs among constraints by transforming the multiple constraints into multi-inequality constraints. The deployment intensity reflects the deployment quality of the network. The more uniform the network deployment is, the closer the network deployment intensity is to 1, which represents a higher deployment quality of the network.

**Figure 4:**
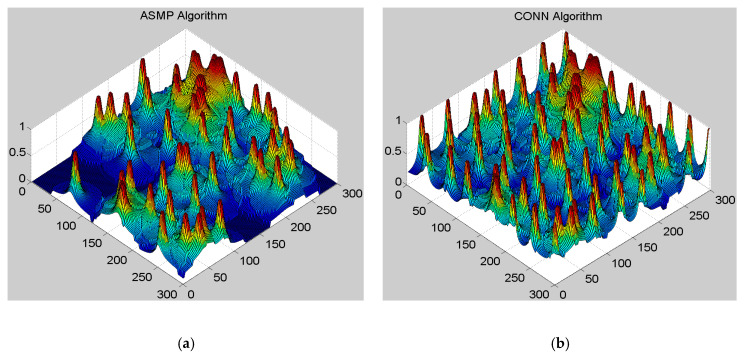
The CONN algorithm is compared with the ASMP algorithm in terms of the deployment intensity. (**a**) The deployment intensity of the ASMP algorithm; (**b**) The deployment intensity of the CONN algorithm.

Figure 4 shows the network deployment intensity for the CONN and ASMP algorithms. It can be seen that in the same network environment, the deployment provided by the CONN algorithm is more uniform than that supplied by the ASMP algorithm. Furthermore, the deployment intensity of the CONN algorithm is higher than that of the ASMP algorithm. The network deployment of the ASMP algorithm has a large number of undeployed regions at the boundary and center, as well as wide areas of overlapping regions. The distribution of a single type of sensor geographically is application-dependent. However, the distribution of multiple types of sensors tends to be a uniform distribution because only a uniform distribution can maximize the use of network resources and reduce the network redundancy. The uniform distribution can improve the quality of the networks as much as possible under the premise of satisfying multiple constraints. In other words, compared with the ASMP algorithm, the CONN algorithm can provide a more reasonable and efficient deployment mechanism. The CONN algorithm can adaptively allocate each neural network module to solve sub-problems. As two neural networks in the CONN algorithm interact and correct each other, the CONN algorithm can provide better deployment solutions.

### 5.4. Comparison with ASMP, EPDS, and OCQ-Max-Fit Algorithms

In another experiment, the ASMP, EPDS, OCQ-Naïve, and OCQ-Max-fit algorithms were compared with the CONN algorithm under a single deployment cost constraint [6,25,26]. The EPDS algorithm is a compound event coverage algorithm based on an environment Pareto-dominated selection strategy. The OCQ-Naive algorithm enumerates all of the feasible deployment schemes and chooses the one that achieves the maximum coverage quality. OCQ-Max-fit enumerates the skyline points of deployment schemes to compute the coverage quality.

In order to evaluate the performance of the CONN algorithm in different environments, the OCQ-Max-fit, ASMP, EPDS, and CONN algorithms were compared in terms of deploying 100 × 100, 200 × 200, …, 1000 × 1000 environments, respectively.

**Figure 5:**
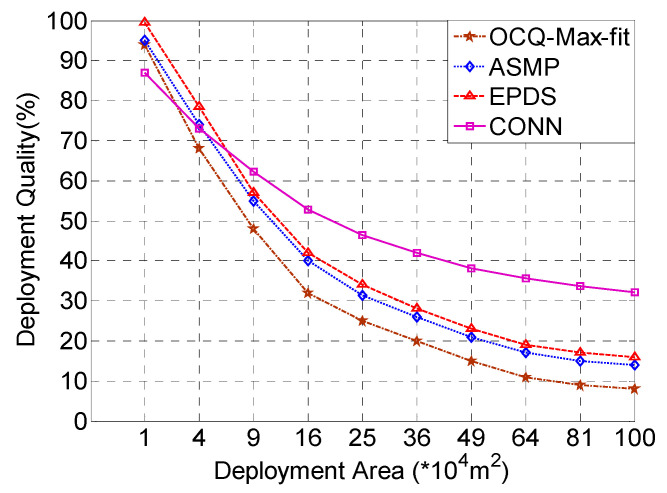
The CONN algorithm is compared with the ASMP, EPDS, and OCQ-Max-fit algorithms in terms of the deployment quality under different deployment environments.

In Figure 5, it can be seen from the experimental results that with the rapid increase in the deployment area, the deployment quality of the OCQ-Max-fit, ASMP, and EPDS algorithms declines rapidly due to a limited budget. When the deployment area is small, the deployment quality of the CONN algorithm is worse than that of the other three algorithms. As the collaborative neural networks continue to learn to obtain better Pareto optimal solutions and optimal confidence coefficients, the deployment quality of the network tends to stabilize and then outperforms the other three algorithms. The CONN algorithm can adaptively allocate each neural network module to solve sub-problems. As two neural networks in the CONN algorithm interact and correct each other, the CONN algorithm can provide better deployment solutions. Therefore, the CONN algorithm can be applied to different deployment areas and provide high-quality deployment solutions, especially for large-area deployments. OCQ-Max-fit enumerates the skyline points of deployment schemes to compute the coverage quality. The deployment schemes which are not dominated by any other deployment scheme are called skyline points. Enumeration methods are very simple and only suitable for a relatively small network scale.

**Figure 6:**
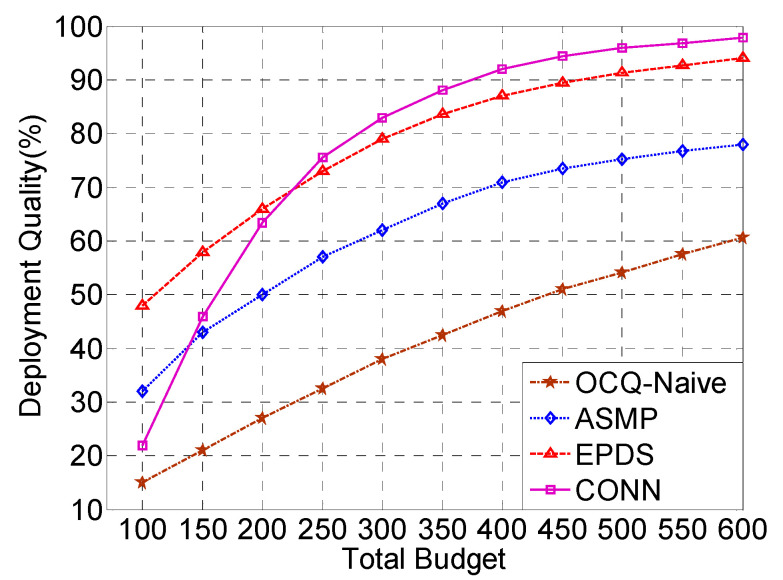
The CONN algorithm is compared with the ASMP, EPDS, and OCQ-Naive algorithms in terms of the deployment quality with an increasing total budget.

Figure 6 shows that as the network budget increases, the quality of deployment increases significantly. When the total budget of the network is low, the deployment quality of the ASMP and EPDS algorithms is better than that of the CONN algorithm. This is due to the fact that the network has fewer deployment schemes when the total budget is lower, which makes the network correction effect worse. The performance of the CONN algorithm is not satisfactory. With the continuous increase of the budget, the deployment quality of the CONN algorithm increases rapidly, soon becoming superior to the other three algorithms. When the network budget is sufficient, the number of sensors that need to be deployed will increase accordingly. When the CONN algorithm deploys a large number of sensors, two neural networks continuously cooperate and correct to obtain better Pareto optimal solutions and optimal confidence coefficients. The global optimal solution is continuously approached to achieve the optimal deployment quality. Concurrently, as the network budget increases, more and more sensors are put into use. The deployment of networks tends to be complicated. The other three algorithms easily fall into a local optimal solution. In particular, the OCQ-Naive algorithm enumerates all of the feasible deployment schemes and chooses the one that achieves the maximum coverage quality. However, it is not necessary to enumerate all of the feasible deployment schemes. Some of the feasible deployment schemes which are certainly not the best ones can be filtered during the enumeration procedure. The CONN algorithm is based on the continuously twice differentiable single-object problem. Therefore, the CONN algorithm is rapidly convergent and globally optimal.

**Figure 7:**
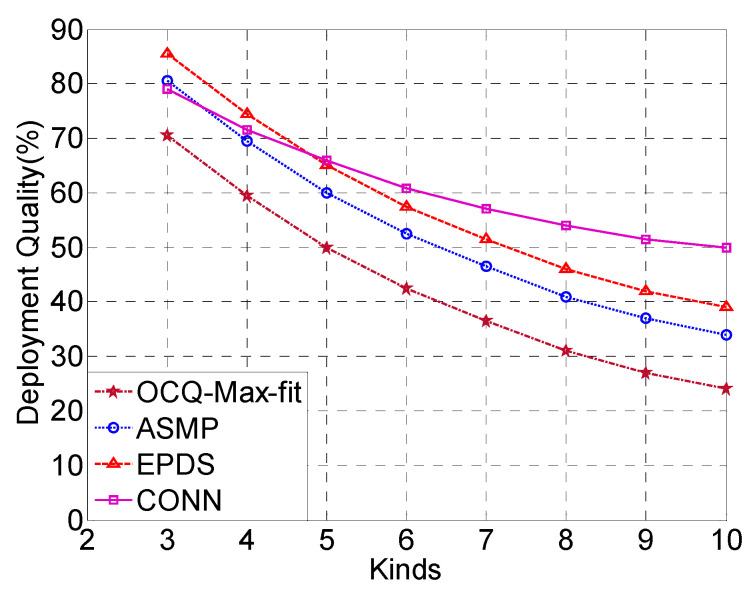
The CONN algorithm is compared with the ASMP, EPDS and OCQ-Max-fit algorithms in terms of the deployment quality with diverse kinds of sensors.

In Figure 7, it can be seen that the deployment quality of the network begins to deteriorate with the increase of the types of sensors. As the compound event is composed of many sub-events, when the total budget is limited, the deployment quality will get worse with the increase in the types of sensors. With fewer types of sensors in the network, the deployment performance of the CONN algorithm is slightly worse than that of the ASMP algorithm and EPDS algorithm. The CONN algorithm does not perform well when the number of sensors is small. As the number of sensors increases in the network, the CONN algorithm adaptively allocates each neural network module to adjust to sub-problems. The network deployment performance of the CONN algorithm is significantly better than the other three algorithms. At the same time, the declining trend of the deployment quality has gradually slowed down. Therefore, it can be seen that the CONN algorithm is more suitable for complicated application scenarios. OCQ-Max-fit enumerates the skyline points of deployment schemes to compute the coverage quality. However, computing the skyline points among all of the feasible deployment schemes is also too costly.

## 6. Conclusions and Future Work

In this paper, a multi-constrained event-driven deployment model based on the maximum entropy function and an event-driven deployment algorithm based on collaborative neural network methods are proposed for a variety of complex monitoring scenarios. The event-driven deployment model effectively solves the problem that the objective function of the min–max method is non-differential. The CONN algorithm can effectively provide an optimal deployment solution according to the different monitoring requirements and network constraints. Moreover, the CONN algorithm effectively overcomes the difficulty of obtaining a large amount of sensor data and environmental information in a complex and dangerous monitoring environment. Finally, the experimental results show that the CONN algorithm can provide effective deployment solutions in a variety of complex multi-constrained network environments.

The multi-constrained event-driven deployment problem has broad application prospects because it is closer to the actual application scenario. In network deployment, especially with the use of camera sensors, privacy protection is an important issue that must be considered. In the future, the issue of privacy protection will be explored in the event-driven deployment of sensor and robot networks.

## Figures and Tables

**Table 1:** The parameters of sensors.

Symbol	Meaning	R/M	T/S	C	Conf
$\alpha_{1}$	Camera Sensor	40	5	40	0.35
$\alpha_{2}$	RFID Sensor	10	2	15	0.20
$\alpha_{3}$	Light Sensor	35	3	30	0.15
$\alpha_{4}$	Bluetooth Sensor	15	4	20	0.05
$\alpha_{5}$	Pyroelectric Sensor	5	1	25	0.10

**Table 2:** The parameters of the experiments.

Case	D/M	T/S	C	MIN	Ratio	Time
1	690	89	810	0.76	42.6%	0.3922 s
2	855	125	1225	0.83	66.2%	0.4086 s
3	1620	256	2175	0.91	89.5%	0.4921 s
4	2075	299	2670	0.98	96.7%	0.8510 s

## References

[1] Dziengel N., Seiffert M., Ziegert M., Adler S., Pfeiffer S., Schiller J. (2016). Deployment and evaluation of a fully applicable distributed event detection system in Wireless Sensor Networks. Ad Hoc Netw..

[2] Zhang P., Nevat I., Peters G.W., Xiao G., Tan H.-P. (2015). Event detection in wireless sensor networks in random spatial sensors deployments. IEEE Trans. Signal. Process..

[3] Guo P., Jiang T., Zhang K. (2012). Novel 2-hop coloring algorithm for time-slot assignment of newly deployed sensor nodes without ID in wireless sensor and robot networks. Comput. Commun..

[4] Wang H., Li Y., Chang T., Chang S., Fan Y. (2018). Event-Driven Sensor Deployment in an Underwater Environment Using a Distributed Hybrid Fish Swarm Optimization Algorithm. Appl. Sci..

[5] Wichmann A., Korkmaz T., Tosun A.S. (2017). Robot Control Strategies for Task Allocation with Connectivity Constraints in Wireless Sensor and Robot Networks. IEEE Trans. Mob. Comput..

[6] Gao J., Li J., Cai Z., Gao H. Composite event coverage in wireless sensor networks with heterogeneous sensors. Proceedings of the IEEE Conference on Computer Communications (INFOCOM).

[7] Koutsougeras C., Liu Y., Zheng R. (2008). Event-driven sensor deployment using self-organizing maps. Int. J. Sens. Netw..

[8] Yang Y., Ambrose A., Cardei M. (2011). Coverage for composite event detection in wireless sensor networks. Wirel. Commun. Mob. Comput..

[9] Gao J., Li J. Model-based approximate event detection in heterogeneous wireless sensor networks. Proceedings of the International Conference on Wireless Algorithms, Systems, and Applications.

[10] Zhou Z., Xing R., Duan Y., Zhu Y., Xiang J. (2015). Event Coverage Detection and Event Source Determination in Underwater Wireless Sensor Networks. Sensors.

[11] Huang H., Savkin A.V., Ding M., Huang C. (2019). Mobile robots in wireless sensor networks: A survey on tasks. Comput. Netw..

[12] Yang C., Chin K.-W. (2016). On nodes placement in energy harvesting wireless sensor networks for coverage and connectivity. IEEE Trans. Ind. Inform..

[13] Costanzo C., Loscrí V., Natalizio E., Razafindralambo T. (2012). Nodes self-deployment for coverage maximization in mobile robot networks using an evolving neural network. Comput. Commun..

[14] Tao D., Wu T.-Y. (2015). A Survey on Barrier Coverage Problem in Directional Sensor Networks. IEEE Sens. J..

[15] Gharaei N., Abu Bakar K., Hashim S.Z.M., Pourasl A.H. (2019). Energy-Efficient Intra-Cluster Routing Algorithm to Enhance the Coverage Time of Wireless Sensor Networks. IEEE Sens. J..

[16] Zhu X., Li J., Chen X., Zhou M. (2017). Minimum Cost Deployment of Heterogeneous Directional Sensor Networks for Differentiated Target Coverage. IEEE Sens. J..

[17] Cao B., Zhao J., Yang P., Yang P., Liu X., Zhang Y. (2019). 3-D Deployment Optimization for Heterogeneous Wireless Directional Sensor Networks on Smart City. IEEE Trans. Ind. Inform..

[18] Chen Z., Teng G., Zhou X., Chen T. (2019). Passive-Event-Assisted Approach for the Localizability of Large-Scale Randomly Deployed Wireless Sensor Network. Tsinghua Sci. Technol..

[19] Janakiram D., Kumar A., Reddy A. Component oriented middleware for distributed collaboration event detection in wireless sensor networks. In Proceeding of the MPAC’05, the 3rd International Workshop on Middleware for Pervasive and Ad-Hoc Computing.

[20] Harrison D.C., Seah W.K.G., Rayudu R. (2018). Coverage Preservation with Rapid Forwarding in Energy-Harvesting Wireless Sensor Networks for Critical Rare Events. ACM Trans. Embed. Comput. Syst..

[21] Zhu W., Cao J., Raynal M. (2018). Energy-Efficient Composite Event Detection in Wireless Sensor Networks. IEEE Commun. Lett..

[22] Wang W., Huang H., He F., Xiao F., Jiang X., Sha C. (2019). An Enhanced Virtual Force Algorithm for Diverse k-Coverage Deployment of 3D Underwater Wireless Sensor Networks. Sensors.

[23] Deng X., Yu Z., Tang R., Qian X., Yuan K., Liu S. (2019). An Optimized Node Deployment Solution Based on a Virtual Spring Force Algorithm for Wireless Sensor Network Applications. Sensors.

[24] Guo J., Jafarkhani H. (2019). Movement-Efficient Sensor Deployment in Wireless Sensor Networks with Limited Communication Range. IEEE Trans. Wirel. Commun..

[25] Zhuang Y., Wu C., Zhang Y., Jia Z. (2017). Compound Event Barrier Coverage in Wireless Sensor Networks under Multi-Constraint Conditions. Sensors.

[26] Zhuang Y., Wu C., Zhang Y., Jia Z. (2017). Compound Event Barrier Coverage Algorithm Based on Environment Pareto Dominated Selection Strategy in Multi-Constraints Sensor Networks. IEEE Access.

[27] Kim W., Yoon W. (2017). Multi-constrained Max–Min Fair Resource Allocation in Multi-channel Wireless Sensor Networks. Wirel. Pers. Commun..

[28] Hasan M.Z., Al-Turjman F., Al-Rizzo H. (2017). Optimized multi-constrained quality-of-service multipath routing approach for multimedia sensor networks. IEEE Sens. J..

[29] Yu J., Lin Y., Wang Y. (2014). Ant-Based Reliable Multi-Constrained Anycast Routing for Sensor Networks. Int. J. Distrib. Sens. Netw..

[30] Akbas A., Yildiz H.U., Ozbayoglu A.M., Tavli B. (2019). Neural network based instant parameter prediction for wireless sensor network optimization models. Wirel. Netw..

[31] Gharghan S.K., Mohammed S.L., Al-Naji A., Abu-AlShaeer M.J., Jawad H.M., Jawad A.M., Chahl J. (2018). Accurate Fall Detection and Localization for Elderly People Based on Neural Network and Energy-Efficient Wireless Sensor Network. Energies.

[32] Jondhale S.R., Deshpande R.S. (2019). Kalman Filtering Framework-Based Real Time Target Tracking in Wireless Sensor Networks Using Generalized Regression Neural Networks. IEEE Sens. J..

[33] Kamel M.S. Neural networks: The state of the art. Proceedings of the 11th International Conference on Microelectronics (IEEE Cat. No.99EX388).

[34] Kamel M.S., Xia Y. (2008). Cooperative recurrent modular neural networks for constrained optimization: A survey of models and applications. Cogn. Neurodynamics.

[35] Xia Y.S. (2004). Further Results on Global Convergence and Stability of Globally Projected Dynamical Systems. J. Optim. Theory Appl..

[36] Ko Y.J., Maystre L., Grossglauser M. Collaborative recurrent neural networks for dynamic recommender systems. Proceedings of the Journal of Machine Learning Research: Workshop and Conference Proceedings.

[37] Chase M., Gilad-Bachrach R., Laine K., Lauter K.E., Rindal P. (2017). Private Collaborative Neural Network Learning. IACR Cryptol. Eprint Arch..

[38] Zhang T., He J., Zhang Y. (2012). Secure Sensor Localization in Wireless Sensor Networks based on Neural Network. Int. J. Comput. Intell. Syst..

[39] Yan X., Cheng H., Zhao Y., Yu W., Huang H., Zheng X. (2016). Real-Time Identification of Smoldering and Flaming Combustion Phases in Forest Using a Wireless Sensor Network-Based Multi-Sensor System and Artificial Neural Network. Sensors.

[40] Nadimi E.S., Jorgensen R.N., Blanes-Vidal V., Christensen S. (2012). Monitoring and classifying animal behavior using ZigBee-based mobile ad hoc wireless sensor networks and artificial neural networks. Comput. Electron. Agric..

[41] Yang J.-B., Sen P. (1996). Interactive tradeoff analysis and preference modelling for preliminary multiobjective ship design synthesis. Syst. Anal. Model. Simul..

[42] Li X. (1992). An entropy-based aggregate method for minimax optimization. Eng. Optim..

[43] Feng Y., Liu H., Zhou S., Liu S. (2008). A smoothing trust-region Newton-CG method for minimax problem. Appl. Math. Comput..

[44] Fliege J., Graña L.M., Svaiter D.B.F. (2009). Newton’s Method for Multiobjective Optimization. Siam J. Optim..

[45] Lin J.G. (2005). On min-norm and min-max methods of multi-objective optimization. Math. Program..

